# The Role of *Microtubule Associated Serine/Threonine Kinase 3* Variants in Neurodevelopmental Diseases: Genotype-Phenotype Association

**DOI:** 10.3389/fnmol.2021.775479

**Published:** 2022-01-12

**Authors:** Li Shu, Neng Xiao, Jiong Qin, Qi Tian, Yanghui Zhang, Haoxian Li, Jing Liu, Qinrui Li, Weiyue Gu, Pengchao Wang, Hua Wang, Xiao Mao

**Affiliations:** ^1^Department of Medical Genetics, Hunan Provincial Maternal and Child Health Care Hospital, Changsha, China; ^2^National Health Commission Key Laboratory for Birth Defect Research and Prevention, Hunan Provincial Maternal and Child Health Care Hospital, Changsha, China; ^3^Department of School of Life Sciences, Central South University, Changsha, China; ^4^Department of Pediatric Neurology, Chenzhou First People’s Hospital, Chenzhou, China; ^5^Department of Pediatrics, Peking University People’s Hospital, Beijing, China; ^6^Medical Genetics Center, Jiangmen Maternity and Child Health Care Hospital, Jiangmen, China; ^7^Cipher Gene LLC, Beijing, China; ^8^Chigene (Beijing) Translational Medical Research Center Co., Ltd., Beijing, China

**Keywords:** *MAST3*, genetics, neurodevelopmental, epilepsy, domain

## Abstract

**Objective:** To prove microtubule associated serine/threonine kinase 3 (*MAST3*) gene is associated with neurodevelopmental diseases (NDD) and the genotype-phenotype correlation.

**Methods:** Trio exome sequencing (trio ES) was performed on four NDD trios. Bioinformatic analysis was conducted based on large-scale genome sequencing data and human brain transcriptomic data. Further *in vivo* zebrafish studies were performed.

**Results:** In our study, we identified four *de novo MAST3* variants (NM_015016.1: c.302C > T:p.Ser101Phe; c.311C > T:p.Ser104Leu; c.1543G > A:p.Gly515Ser; and c.1547T > C:p.Leu516Pro) in four patients with developmental and epileptic encephalopathy (DEE) separately. Clinical heterogeneities were observed in patients carrying variants in domain of unknown function (DUF) and serine-threonine kinase (STK) domain separately. Using the published large-scale exome sequencing data, higher CADD scores of missense variants in DUF domain were found in NDD cohort compared with gnomAD database. In addition, we obtained an excess of missense variants in DUF domain when compared autistic spectrum disorder (ASD) cohort with gnomAD database, similarly an excess of missense variants in STK domain when compared DEE cohort with gnomAD database. Based on Brainspan datasets, we showed that *MAST3* expression was significantly upregulated in ASD and DEE-related brain regions and was functionally linked with DEE genes. In zebrafish model, abnormal morphology of central nervous system was observed in *mast3a/b* crispants.

**Conclusion:** Our results support the possibility that *MAST3* is a novel gene associated with NDD which could expand the genetic spectrum for NDD. The genotype-phenotype correlation may contribute to future genetic counseling.

## Introduction

*Microtubule associated serine/threonine kinase 3* [*MAST3* (MIM 612258)] gene belongs to microtubule-associated serine/threonine kinase (MAST) family which harbors conserved protein domains including a central microtubule-associated serine-threonine kinase domain (STK) flanked by domain of unknown function (DUF) (Hain et al., 2014). *MAST3* is selectively expressed in brain especially in cerebral cortex (Garland et al., 2008) and mediated vital neuronal functions, such as neuronal survival, neurite outgrowth, and others (Delhommel et al., 2015; Khan et al., 2019).

In *MAST* gene family, *MAST1* gene has been reported to cause neurodevelopmental diseases (NDD). The representative clinical phenotypes of the patients are mega-corpus-callosum syndrome with cerebellar hypoplasia and cortical malformations (MCC-CH-CM) (MIM:618273) (McMichael et al., 2015; Tripathy et al., 2018; Ben-Mahmoud et al., 2020; Rodriguez-Garcia et al., 2020). *MAST2-4* genes have never been reported to be associated with neurological disorders until 2021. Spinelli et al. (2021) reported that patients with *de novo* variants in STK domain of *MAST3* gene showed concomitant developmental and epileptic encephalopathy (DEE) phenotypes (Spinelli et al., 2021).

In our study, we identified four *de novo* heterozygous variants in *MAST3* gene in four NDD patients using trio exome sequencing (trio ES). Genotype-phenotype correlations were observed. Together with bioinformatic analyses and functional studies on zebrafish model, our study indicated that *MAST3* variants may be related to variable neurodevelopmental phenotypes from intellectual disability (ID) with epilepsy to ID with autistic spectrum disorder (ASD).

## Materials and Methods

### Trio Exome Sequencing

Genomic DNA from peripheral blood leukocytes of four trios were extracted using the Qiagen DNA Blood Midi/Mini Kit (Qiagen GmbH, Hilden, Germany). DNA was then captured using the IDT xGen Exome Research Panel (Integrated DNA Technologies, San Diego, CA, United States) and was sequenced on Novaseq 6000 platform (Illumina, San Diego, CA, United States). Sequencing reads were aligned to the human reference genome (UCSC hg19/GRCh37). Bioinformatic analyses were performed according to the standard protocol of Ulintz et al. (2019). Human population databases, such as gnomAD, ExAC, and 1000 genomes were used for variant filtration. *In silico* prediction algorithms, such as SIFT, Polyphen-2, LRT, and MutationTaster were used for variant pathogenicity. GERP++, phyloP, phastCons, and SiPhy were used for variant conservation prediction. Sanger sequencing was performed for variant validation.

### Bioinformatics and Statistics

#### Burden Analysis

ANNOtate VARiation (ANNOVAR) was used to annotate all *MAST3* variants (Wang et al., 2010). Excess of *MAST3 de novo* variants was analyzed using two statistic models (DenovolyzeR and CH model). Briefly, we derived the expected number of *de novo* events in a given population based on the mutability of the *MAST3* and the number of probands sequenced. Then, we compared the observed number of *de novo* variants against expectation using Poisson framework (DenovolyzeR) or binormal model (CH model) (O’Roak et al., 2012; Lal et al., 2020). Correction for multiple tests was performed using Bonferroni method.

The *MAST3* expression pattern in the brain development: RNA-seq data in different developmental stages (from 8 post-conceptional weeks to 40 years) from multifarious brain regions were obtained from Brainspan.^1^ RNA expression was normalized to reads per kilobase million (RPKM). Univariate linear regression was applied by using lm() function in R^2^ for analyzing *MAST3* expression mode in temporal cortex (TC) and dorsolateral prefrontal cortex (DFC).

#### Co-expression Analysis

A gene with RPKM >0.5 in 80% of all developing cortex tissues was regarded as a cortex-expressed gene. In total, 50 known genes associated with dominant DEE syndromes were defined as known DEE gene set (Heyne et al., 2018). Furthermore, 143 known NDD genes combined 50 known DEE genes with NDD genes identified in previous research (Heyne et al., 2018). Two gene sets were both applied to calculate Spearman’s correlation coefficient with all cortex-expressed genes. Next, the mean of Spearman’s correlation coefficient of each cortex-expressed gene in known DEE gene set or NDD gene set was computed. Percentile of average correlation coefficient between *MAST3* and DEE gene set or NDD gene set was acquired.

### Zebrafish Studies

#### Zebrafish Maintenance

Adult zebrafish was maintained in tanks with circulating water at 28°C on a 14/10 h light/dark cycle and fed two times a day. Zebrafish embryos were obtained by mating adult fish through standard methods (Avdesh et al., 2012). Larvae were raised in embryo media consisting of 0.03% Instant Ocean and 0.0002% methylene blue in reverse osmosis-distilled water. Transgenic line [*Tg* (*HuC:eGFP*)](Park et al., 2000) with neuron-expressing enhanced green florescent protein (eGFP) were used. All procedures were performed in accordance with the Guide for the Care and Use of Animals (National Research Council, 2011) and the guidelines and regulations of Cipher Gene, LLC.

#### Gene Editing and Tracking of Indels by Decomposition Assessment

The zebrafish genome has two *MAST3* orthologs: *mast3a* (ENSDARG00000061725) and *mast3b* (ENSDARG00000086505). Single guide RNA (sgRNA) targets were identified using the CHOPCHOP online tool and were obtained from GenScript. Four sgRNAs designed for each gene are as follows (PAM sequence in lowercase): cccACCCCAGATGACCTCAATCG, cccCAGATGACCTCAAT CGCCTC, cccTCTCGGTTCCATCCTCGCTC, cctCTCGGTT CCATCCTCGCTCC for *mast3a*, and ccaCCAGCTTCCATA CCAGCCCA, ccgAAGTTCGGAGAGCATGACTG, cctCCAC GTTTATAAGACCCCGG, cccCGGTCACGTAGCCTTAGGTA for *mast3b*. The ortholog evaluation is conducted using the online tool-DIOPT Ortholog Finder. The protein identities between human MAST3 and zebrafish ortholog genes are 62% for mast3a and 63% for mast3b. The protein identity between zebrafish paralogs mast3a and mast3b is 70%. To generate mutagenesis in each targeted gene, fertilized embryos (1–2 cell stage) were injected with ∼2 nl CRISPR complexes composed of four sgRNAs (90 ng/μl for each sgRNA) and Cas9 protein (200 ng/μl). At 24 h after injection, few embryos from the injected group were pooled and sanger sequenced to verify the mutagenesis efficacy using the tracking of indels by decomposition (TIDE) online tool (Brinkman et al., 2014). *Post hoc* genotyping was performed after phenotypic studies. Individual larvae were collected for sequencing and TIDE analysis was used to confirm the mutagenesis. Larvae with TIDE efficacy <5% were excluded from phenotypic data analysis.

#### Imaging and Morphological Measurement

At six dpf, *Tg* (*HuC*: *eGFP*) larvae were placed in a customized mini-well (5 mm diameter and 1 mm depth) plate (one fish/well) with dorsal side up for bright-field and florescent imaging. Images were acquired with a Touptek CCD camera and Nikon SMZ800N stereo fluorescence microscope with × 1.5 magnification for bright-field imaging and × 4 magnification for florescent imaging. Images were then analyzed in Fiji (ImageJ) for body length and central nervous system (CNS) area measurements.

#### Statistics

Statistical analyses were performed with Prism 8 (GraphPad Software, CA, United States), using unpaired *t*-test for qualitative data. Significance for all tests was defined as *p < 0.05; ^**^p < 0.01; and ^***^p < 0.001.

## Results

### Clinical Presentations of Patients

The clinical features of patient 1–4 are shown in Table 1. Patient 1 is a 6-year-old male at present who was born at full term with normal pregnancy. At present, patient 1 has reached a normal motor developmental milestone. The 6-year-old male rarely communicate and have trouble learning to count. Patient 1 was fascinated by spinning wheels of a car, playing with hands, and repeating words of people.

**TABLE 1:** Summary of genotype and phenotype information for NDD in individuals with *Microtubule associated serine/threonine kinase 3* variants.

Case index	1	2	3	4
Variant (NM_015016.1)	c.302C > T:p.Ser101Phe	c.311C > T:p.Ser104Leu	c.1547T > C:p.Leu516Pro	c.1543G > A:p.Gly515Ser
ACMG classification	Likely pathogenic (PS2, PM1, PM2)	Likely pathogenic (PS2, PM1, PM2 PP3)	Likely pathogenic (PS2, PM1, PM2)	Likely pathogenic (PS2, PM1, PM2)
Protein domain the variant located	DUF	DUF	STK	STK
Age at last examination (years)	Six	four and a half	Ten	twelve
Age at onset-seizure	NA	NA	3 months	1 year and 11 months
Gender	male	Male	Male	male
Global developmental delay/intellectual disability (diagnosed by Gessel or WAIS)	Yes	Yes	Yes	yes
Epilepsy type	NA	NA	LGS	GTCS
Epilepsy controlled	NA	NA	No	yes
ASD (diagnosed by ABC, CARS)	Yes	Yes	No	no
Hypotonia	No	No	Yes	no
Brain MRI	normal	normal	Normal	normal
EEG	NA	NA	15–25 Hz low amplitude fast waves and complex slow waves burst for 1.2 s, followed by diffuse voltage reduction for 4 s and a large number of multifocal and 1.5–2.5 Hz high amplitude spike-slow waves with sharp waves and slow waves (3 months); 15–25 Hz spikes burst intermittently for 2.5 s on the background of diffuse slow waves (2 years);20–25 Hz spikes burst for 4.5 s (2 years and 3 months)	spikes in bilateral central, parietal and temporal areas of brain

*PS2, de novo (both maternity and paternity confirmed) in a patient with the disease and no family history; PM1, Located in a mutational hot spot and/or critical and well-established functional domain (e.g., active site of an enzyme) without benign variation; PM2, Absent from controls (or at extremely low frequency if recessive) in Exome Sequencing Project, 1000 Genomes Project, or Exome Aggregation Consortium; PP3, Multiple lines of computational evidence support a deleterious effect on the gene or gene product (conservation, evolutionary, splicing impact, etc.); WAIS, Wechsler Adult Intelligence Scale; ABC, Autistic Behavior Checklist; CARS, Childhood Autism Rating Scale.*

Patient 2 is a four year and a half male who was born at full term without abnormality in pregnancy. At present, patients 2 is easy to fall when running and cannot get dressed. This patient does not want to play with peers and lacks eye contact.

Patient 3 is a 10-year-old male child who was born at full term with normal pregnancy. The patient 3 had seizures at the age of 3 months without inducement. Frequent nodding about 10 times a day was observed. Patient 3 began to present typical “nodding and holding ball” movements soon, accompanied by loss of consciousness, cyanosis of lips, and face. The boy was diagnosed as “infantile spasms” and given topiramate as anti-epileptic therapy. At the age of 2 years, the “nod and holding ball” movements disappeared gradually, and a new attack form appeared. Eyes staring, rigidity, and contraction of limbs, accompanied by loss of consciousness, mild cyanosis of face and lips were observed. Suspected Lennox-Gastaut syndrome (LGS) was diagnosed. After 3 months, another new attack form appeared that the patient showed sudden head tilting forward, mouth opening accompanied by salivation. The patient was diagnosed as “LGS.” After 1 year of treatment with clonazepam, the patient still had many atypical absence seizures per day. The patient presented with global developmental delay and could not walk or speak words. No other neurological abnormality was observed.

Patient 4 is a 12-year-old male who was born at full term without abnormality in pregnancy. At the age of 1 year and 11 months, without inducement, generalized tonic-clonic seizure epilepsy (GTCS) was observed. Anti-epileptic drug levetiracetam was given to the patient and the seizures were relieved. At present, motor development is normal but language development is delayed.

Hence, it is reasonable to speculate that due to different protein domains in which the variants are located, these four patients showed different clinical features.

### Variant Predictions

**FIGURE 1:**
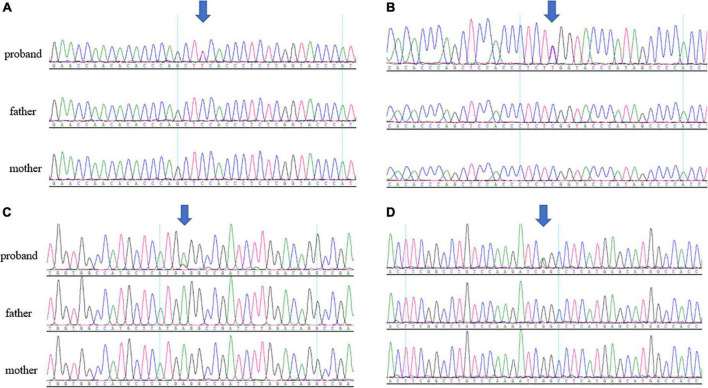
**(A)** Sanger sequencing results of c.302C > T in patient 1 trio. **(B)** Sanger sequencing results of c.311C > T in patient 2 trio. **(C)** Sanger sequencing results of c.1547T > C in patient 3 trio. **(D)** Sanger sequencing results of c.1543G > A in patient 4 trio. The arrows pointed to the location of the variants.

**FIGURE 2:**
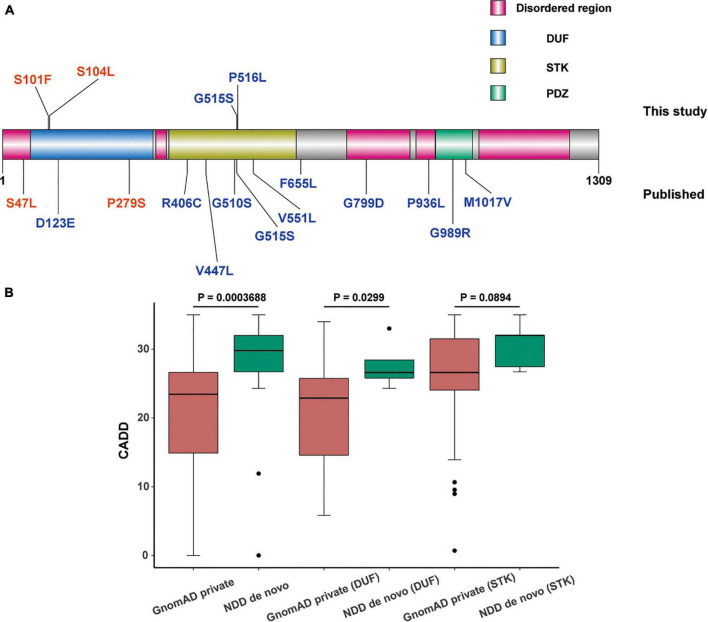
Distribution and comparison of pathogenicity of missense mutations in domain of unknown function (DUF) and serine-threonine kinas (STK) domain of *microtubule associated serine/threonine kinase 3* (*MAST3*). **(A)** Schematic representation of missense variations in *MAST3*. Variations above and under the protein domain graph are originated from this study and published research, respectively. The colors in the *MAST3* protein represented different protein domains (pink: disordered region, blue: DUF domain, yellow: STK domain, and green: PDZ domain). The different colors of the variants showed different diseases (red, ASD; blue, DD). **(B)** Box plot of CADD of *de novo* (neurodevelopmental diseases, NDD)/private (GnomAD) missense mutations in DUF or STK domain. Higher CADD scores of missense variants in total and in DUF region of *MAST3* were found in NDD cohort compared with that in gnomAD database (*p* = 0.0003688, 0.0229, Wilcoxon test). Significance was lost when the same analysis was performed for STK domain variants (*p* = 0.0894, Wilcoxon test).

Four novel *de novo MAST3* variants were identified in proband 1–4 separately using trio-ES and Sanger validation (Table 1 and Figure 1). These variants are classified as likely pathogenic by ACMG guidelines. They are absent from human population databases gnomAD, ExAC, and 1000 genomes. They are predicted to be deleterious by SIFT, Polyphen-2, LRT, MutationTaster algorithms and predicted to be conserved by GERP++, phyloP, phastCons, and SiPhy algorithms. These variants lied in protein domains DUF and STK in *MAST3* (Figure 2A).

### Bioinformatic Analysis

#### *De novo Microtubule Associated Serine/Threonine Kinase 3* Missense Variants in Domain of Unknown Function and Serine-Threonine Kinase Domain Exhibit Excess in Neurodevelopmental Diseases

In our study, we found a significant enrichment of *MAST3* variants (we did not include two missense variants without detailed data) (denovolyzeR: *p* = 0.00152, Bonferroni = 1; CH-model: *p* = 0.001994, Bonferroni = 1) *via* analysis of *de novo* variants by two statistic models. However, the significance did not exist after we performed a genome-wide Bonferroni correction. By combining published NDD studies with large cohorts, fifteen *de novo* missense variants (we did not include two missense variants without detailed data) were obtained from 36,347 probands (Table 2 and Figure 2A). We observed significant results in two models (denovolyzeR: *p* = 4.07E-06, Bonferroni = 0.0798; CH-model: *p* = 2.051E-05, Bonferroni = 0.40236518). However, statistically non-significant results were found after correction in the scope of genome (corrected for ∼19,000 genes).

**TABLE 2:** Summary of *Microtubule associated serine/threonine kinase 3 de novo* missense variants identified in neurodevelopmental disorders.

Index	Sample ID	gDNA change (chr19, hg19)	Function	Coding change	Protein change	SIFT	Polyphen-2	CADD	Inheritance	gnomAD (non-neuro)	PMID	Cohort size	Primary diagnosis
1	UK10K_SKUSE5080236	g.18232563C > T	Missense	c.140C > T	p.47S > L	D	D	32	*de novo*		31981491	3,899	ASD
2	9Y0069	g.18233551C > T	Missense	c.302C > T	p.101S > F	D	D	33	*de novo*		This study	585	ASD
3	DDN20002957	g.18233560C > T	Missense	c.C311T	p.S104L	D	D	26.3	*de novo*		This study (co-operation)	30,000	ASD
4	DDD4K.02825	g.18234083C > G	Missense	c.369C > G	p.123D > E	T	P	24.3	*de novo*		28135719	4,293	DD
5	1-0998-003	g.18235153C > T	Missense	c.835C > T	p.279P > S	D	D	26.9	*de novo*		28263302	1,625	ASD
6	DDD13k.01676	g.18241383C > T	Missense	c.1216C > T	p.406R > C	D	D	35	*de novo*		33057194	25,945	DD
7	117791	g.18241506G > C	Missense	c.1339G > C	p.447V > L	T	P	27.5	*de novo*		33057194	25,945	DD
8	DDD13k.00098	g.18245432G > A	missense	c.1528G > A	p.510G > S	D	D	32	*de novo*		33057194	25,945	DD
9	76597	g.18245447G > A	Missense	c.1543G > A	p.515G > S	D	D	32	*de novo*		33057194	25,945	DD
10	DD18006823	g.18245447G > A	Missense	c.G1543A	p.515G > S	D	D	32	*de novo*		This study (co-operation)	30,000	EE
11	9Y3441	g.18245451T > C	Missense	c.1547T > C	p.516L > P	D	P	26.7	*de novo*		This study	585	EE
12	96317	g.18245660G > T	Missense	c.1651G > T	p.551V > L	D	P	27.4	*de novo*		33057194	25,945	DD
13	97813	g.18248126T > C	Missense	c.1963T > C	p.655F > L	D	P	29.8	*de novo*		33057194	25,945	DD
14	2677	g.18254716G > A	Missense	c.2396G > A	p.799G > D	T	B	0.028	*de novo*		33057194	25,945	DD
15	DDD13k.00479	g.18255894C > T	Missense	c.2807C > T	p.936P > L	D	D	33	*de novo*	0.00007704	33057194	25,945	DD
16	DDD13k.03362	g.18256565G > A	Missense	c.2965G > A	p.989G > R	D	D	33	*de novo*		33057194	25,945	DD
17	3099	g.18256649A > G	Missense	c.3049A > G	p.1017M > V	T	B	11.92	*de novo*	0.00001239	33057194	25,945	DD

*Isoform, NM_015016.2; ASD, Autism Spectrum Disorder; DD, Developmental Disorder; EE, Epileptic Encephalopathy; T, Tolerable; P, Possibly_damaging; B, Benign; D, Deleterious.*

Interestingly, in our cohort, four *de novo MAST3* missense variants lie in DUF or STK domain separately, so we expected to further explore the domain-related clinical heterogeneity. For DUF domain (58–311 aa), higher CADD scores of missense variants in *MAST3* were found in NDD cohort compared with that in gnomAD database (*p* = 0.0229, Wilcoxon test) (Figure 2B). Significance was lost when the same analysis was performed for STK domain variants (366–645 aa) (*p* = 0.0894, Wilcoxon test) though it was closed to the threshold of statistical significance (Figure 2B). More importantly, we obtained an excess of missense variant in DUF domain when only consider ASD cohort compared with gnomAD database [2 probands in 2,210 ASD individuals vs. 48 carriers in 125,748 gnomAD samples, odds ratio (*OR*) = 2.37, *p* = 0.2139, Fisher’s exact test]. Similarly, a higher *OR* was found in STK domain when only consider DEE cohort compared with gnomAD database (1 probands in 585 individuals vs. 51 carriers in 125,748 gnomAD samples, *OR* = 4.21, *p* = 0.2147, Fisher’s exact test). Above insignificant *p*-value may be due to insufficient number of individuals carrying *MAST3* variants. These results supported our clinical data to some extent.

#### Spatio-Temporal Modes of *Microtubule Associated Serine/Threonine Kinase 3*

**FIGURE 3:**
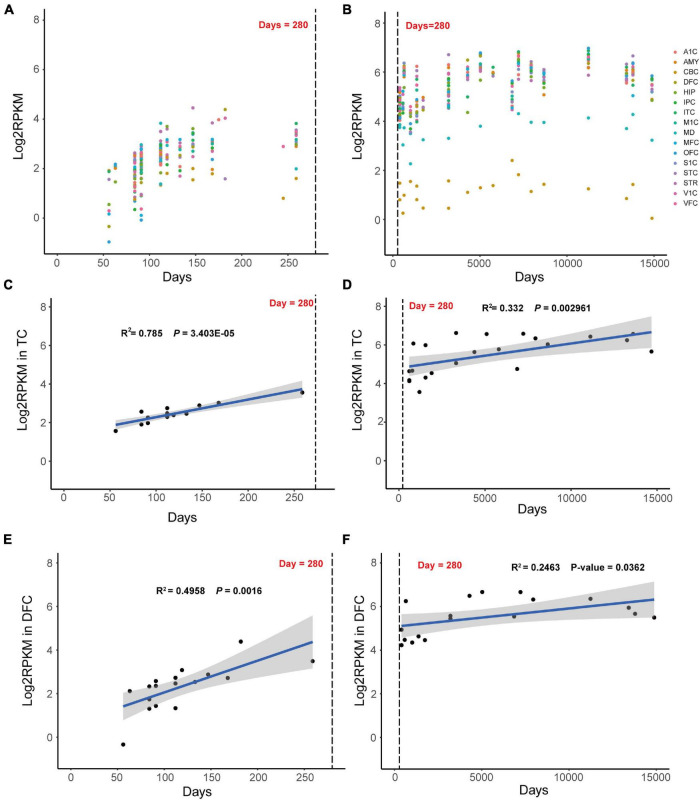
Dynamic expression mode of *MAST3* in human development brains. **(A,B)** Expression of *MAST3* during brain development periods in different brain regions. The *x*-axis is the age of samples in days and *y*-axis is the log2-transformed RPKM of *MAST3*. A1C, primary auditory cortex; AMY, amygdaloid complex; CBC, cerebellar cortex; DFC, dorsolateral prefrontal cortex; HIP, hippocampus; IPC, inferior parietal cortex; ITC, inferolateral temporal cortex; M1C, primary motor cortex; MD, mediodorsal nucleus of thalamus; MFC, medial prefrontal cortex; OFC, orbital frontal cortex; S1C, primary somatosensory cortex; STC, superior temporal cortex; STR, striatum; V1C, primary visual cortex; VFC, ventrolateral prefrontal cortex. The dashed line indicates the birthday. **(C,D)** Expression of *MAST3* in the TC region. **(E,F)** Expression of *MAST3* in the DFC region. Univariate regression analysis was applied in prenatal brains (left) and post-natal brains (right), separately. Blue line represents the regression line and the gray region indicates 95% CI. The text shows the *R*^2^ and *P*-value of regression.

To elucidating the key role of *MAST3* in developing brain, RNA sequencing data from BrainSpan was used to find out the spatio-temporal expression pattern of *MAST3*. We found a time-dependent upregulation *MAST3* expression in fetal brain (Figure 3A). Differently, high expression level was observed during post-birth periods in most brain regions (Figure 3B). To reveal a dynamic change of *MAST3* in DEE-related and ASD-relevant brain regions during brain development, such as TC and DFC (Ives-Deliperi and Butler, 2021; Jassim et al., 2021), a univariate linear regression analysis was applied. In TC and DFC, expression of *MAST3* was significantly upregulated during the period of embryonic development (TC: *R*^2^ = 0.785 and *p* = 3.403E-05, DFC: *R*^2^ = 0.4958 and *p* = 0.0016) than that during the post-natal period (TC: *R*^2^ = 0.332 and *p* = 0.002961, DFC: *R*^2^ = 0.2463 and *p* = 0.0362) (Figures 3C–F). These data implicated a potential role of *MAST3* during embryonic developing periods.

#### Co-expression of *Microtubule Associated Serine/Threonine Kinase 3* With Well-Established DEE/Neurodevelopmental Diseases Genes

Microtubule associated serine/threonine kinase 3 is a member of Microtubule Associated Serine/Threonine Kinase family. Regrettably, the biological function of MAST3 in NDD still remains unknown. PTEN which interacts with MAST3 from STRING database was found to be related to DEE, ASD, etc. (Hobert et al., 2014; Marchese et al., 2014).

**FIGURE 4:**
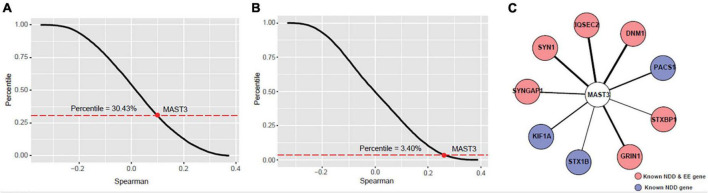
Co-expression analyses of microtubule associated serine/threonine kinase 3 (MAST3) with EE and NDD genes. **(A,B)** The percentile of average correlation coefficient for all developing cortex expressed genes with NDD **(A)** or EE **(B)** genes. The average percentile of MAST3 was marked. **(C)** Interaction network contains nine highly co-expressed EE/NDD genes (Spearman’s correlation coefficient > 0.7) with MAST3.

We further explored the potentially biologic connection of *MAST3* with DEE and NDD genes using gene co-expression analysis. We wondered whether *MAST3* is highly co-expressed with DEE or NDD genes compared with other cortex-expressed genes. Spearman’s correlation coefficients of all protein-coding genes with well-established DEE and NDD gene sets were calculated. *MAST3* was strongly correlated with known DEE genes (Percentile = 3.40%) compared with NDD genes (Percentile = 30.43%) (Figures 4A,B). In total, nine DEE/NDD genes were highly correlated with *MAST3* (Spearman’s correlation coefficient > 0.7) (Figure 4C). Above analysis uncovered the potential relationship between *MAST3* and DEE/NDD genes.

### *In vivo* Studies

We created zebrafish models with CRISPR/cas9-induced disruptions in *mast3a* or *mast3b*, two orthologs of human *MAST3*, to investigate the role of *MAST3* in neurodevelopment *in vivo*. The zebrafish F0 CRISPR (crispants) recapitulated the characteristic phenotypes in human NDD-abnormal brain morphology.

**FIGURE 5:**
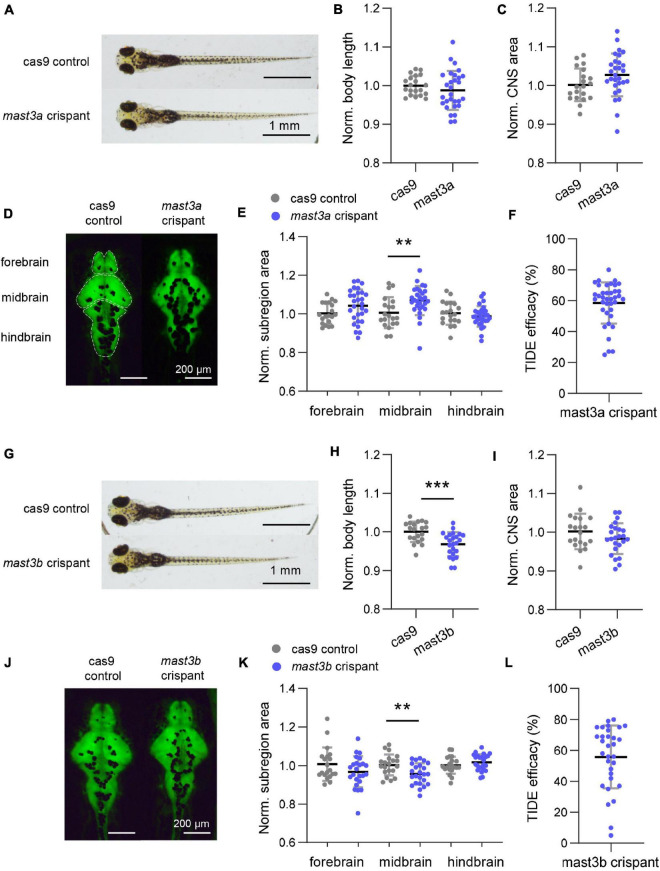
Disruption of zebrafish *mast3a* and *mast3b* led to abnormal central nervous system (CNS) morphology. **(A)** Representative bright-field imaging of larval zebrafish at six dpf (dorsal view). Top, cas9 injected control; bottom, *mast3a* F0 CRISPR (crispant). **(B)** Measurements of body length in cas9 injected control (*n* = 20 fish) vs. *mast3a* crispant (*n* = 29 fish). Data were normalized to the average body length of cas9 control group. **(C)** Normalized CNS area in cas9 injected controls vs. *mast3a* crispants. Data were corrected by the normalized body length of individual larvae. **(D)** Representative imaging of HuC: eGFP expressed larval zebrafish shows CNS fluorescence pattern at six dpf (dorsal view). Left, cas9 injected control; right, *mast3a* crispant. White dash line highlights the CNS subregions, forebrain, midbrain, and hindbrain, for measurement. **(E)** Normalized CNS subregion area in cas9 injected control vs. *mast3a* crispant. Data were normalized to the average of each subregion area of cas9 control group, and then corrected by the normalized body length of individual larvae (gray dot, cas9 control; blue dot, *mast3a* crispant). **(F)** CRISPR efficacy calculated *via* TIDE method of individual *mast3a* crispant used in phenotypic study (*n* = 38 fish). **(G–L)** Data from *mast3b* crispant study (for imaging study, *n* = 20 fish for cas9 control, and *n* = 24 fish for *mast3b* crispant; *n* = 31 crispant for TIDE efficacy verification). Scale bars as indicated in the figure. Error bars indicate SD. Statistical significance is indicated as **p < 0.01, and ***p < 0.001.

At six dpf, *mast3a* crispants showed normal body length (Figures 5A,B; *n* = 20 and 29 fish for cas9 control and *mast3a* crispant, respectively; unpaired *t*-test, *p* = 0.3208). Although no significant difference was observed in the whole CNS area (Figures 5C,D; unpaired *t*-test, *p* = 0.0844), the midbrain area was significantly increased in *mast3a* crispant vs. cas9 controls (Figure 5E; unpaired *t*-test, *p* = 0.0726, 0.0072, and 0.3123 for forebrain, midbrain, and hindbrain area, respectively). *Mast3b* crispants presented significantly decreased body length (Figures 5G,H; *n* = 20 and 24 fish for cas9 control and *mast3b* crispant, respectively; unpaired *t*-test, *p* = 0.0007). Although no statistically significant difference was observed in the whole CNS area (Figures 5I,J; unpaired *t*-test, *p* = 0.1558), the midbrain area was significantly decreased in *mast3b* crispants (Figure 5K; unpaired *t*-test, *p* = 0.1268, 0.0073, and 0.2807 for forebrain, midbrain, and hindbrain area, respectively). Electrophysiology and behavior studies were also conducted. No electrographic epileptiform activity or abnormal locomotion behavior was observed in the crispant larvae (*n* = 38 and 31 fish were tested for *mast3a* and *mast3b* crispants, respectively). Figures 5F,L showed the TIDE efficacy of the crispant larvae for phenotypic analysis. These results suggest that, as in humans, zebrafish *mast3a/b* is critical for brain development and possibly neuronal function.

## Discussion

Microtubule-associated serine/threonine kinase protein family has been proved to be highly expressed in brain and was involved in multiple critical neuronal functions (Jing et al., 2020). *De novo* variants in STK domain of *MAST3* gene has been reported to be candidate variants for Rett syndrome-like phenotypes (Iwama et al., 2019) and associated with DEE recently (Spinelli et al., 2021). In our study, combining genetic testing with bioinformatic analysis and functional studies, we reported the role of *MAST3* variants in NDD and its genotype-phenotype correlations.

We identified four *de novo* heterozygous *MAST3* variants in four unrelated patients with NDD. Although all patients showed an NDD phenotypic profile, patient 1 and 2 showed ID with ASD while patient 3 and 4 showed ID with epilepsy. Variants in *MAST3* exhibited heterogeneous clinical presentations, which probably due to different domains the variants located in Figure 2A.

These genotype-phenotype relationships were also supported by bioinformatic analysis based on large NDD cohorts. For DUF domain, higher CADD scores of missense variants for *MAST3* were found in NDD cohort compared with gnomAD database. More importantly, we obtained an excess of missense variants in DUF domain when compared ASD cohort with gnomAD database and similarly an excess of missense variants in STK domain when compared DEE cohort with gnomAD database. More patients with *MAST3* variants in DUF domain may yield a significant *p*-value. By applying univariate linear regression analysis using RNA sequencing data from BrainSpan, we found that in ASD-relevant brain region DFC and DEE-related brain region TC (Ives-Deliperi and Butler, 2021; Jassim et al., 2021), the expression of *MAST3* was significantly upregulated during brain development. From the co-expression analysis, nine DEE/NDD genes (*SYNGAP1, SYN1, IQSEC2, DNM1, PACS1, STXBP1, GRIN1, STX1B, and KIF1A*) were highly correlated with *MAST3*. Known NDD and/or EE genes, such as *IQSEC2, DNM1*, and *STX1B*, have all been proved to play a role in vital synaptic functions, such as synaptic vesicle recycling, excitatory synaptic transmission, and calcium-dependent synaptic vesicle release in neurodevelopment (Smirnova et al., 1996; Boumil et al., 2010; Brant et al., 2021). The mutants of these genes could cause synaptic deficit and be associated with NDD and/or EE. The *MAST3* mutations might affect the normal interactions with the nine DEE/NDD genes, cause neuronal dysfunctions thus leading to neurodevelopmental phenotypes. Although there was no supporting evidence to these functional interactions, our study provided a clue for further research.

From the functional perspective, the variants located in the domains might influence the catalytic activity of MAST3 (Iwama et al., 2019), thus affecting its interaction with different targeted proteins involved in neuronal functions (Valiente et al., 2005; Delhommel et al., 2015). It has been reported that MAST3 protein may act synergically with PTEN to moderate signal pathways in neuronal survival, neurite outgrowth, and regeneration (Khan et al., 2019). MAST3 protein could also interact with seizure related transcription factors, zif268, c-fos, c-jun, etc. (Saffen et al., 1988; Xiao et al., 1998). According to our bioinformatic analysis, *MAST3* gene was highly co-expressed with DEE or NDD genes compared with cortex-expressed other genes.

Due to the teleost-specific genome duplication (Meyer and Schartl, 1999; Howe et al., 2013), certain zebrafish gene pairs (ohnologs) have acquired different expression patterns or functions, which could be redundant function, new function, sub-function, or pseudo-function. As a result, it is not unusual to see in zebrafish study that disruptions of a/b copy of a gene show different phenotypes (Force et al., 1999; Kleinjan et al., 2008; Shin et al., 2012; Grone et al., 2016; Holt et al., 2019). In this work, brain morphological abnormality was observed in both zebrafish mast3a and mast3b crispants, so we concluded that zebrafish mast3a and mast3b are critical for brain development. Further study will be needed to elucidate how exactly these genes contribute to the brain development. In addition, human patients carrying *MAST3* missense mutations demonstrates various phenotypes, such as macrocephaly and microcephaly, which may indicate the pathological complexity (Spinelli et al., 2021). The functional study based on zebrafish model also provided evidence for *MAST3* pathogenicity by recapitulating the neurological phenotype of DEE–abnormal brain morphology. Together with the genetic and bioinformatic analysis, our work may confirm the pathogenicity of *MAST3* gene in NDD. However, further functional research on specific neurodevelopmental defects and larger cohorts were needed to elucidate the mechanisms underlying the genotype-phenotype correlation.

## Conclusion

In conclusion, we first described the genotype-phenotype correlations of *MAST3* gene in NDD. The variants of DUF domain contributed to NDD with a core ASD phenotype while variants of STK contributed to NDD with a core epilepsy phenotype, therefore, conducive to future genetic counseling of *MAST3*.

## Data Availability Statement

The datasets for this article are not publicly available due to concerns regarding participant/patient anonymity. Requests to access the datasets should be directed to the corresponding authors.

## Ethics Statement

The studies involving human participants were reviewed and approved by the Ethics Committee of Maternal and Child Health Hospital of Hunan Province (2020-S003). Written informed consent to participate in this study was provided by the participants’ legal guardian/next of kin. Written informed consent was obtained from the individual(s), and minor(s)’ legal guardian/next of kin, for the publication of any potentially identifiable images or data included in this article. The animal study was reviewed and approved by the Ethics Committee of Maternal and Child Health Hospital of Hunan Province (2020-S003).

## Author Contributions

XM and HW designed the research. LS and NX wrote the manuscript. YZ and HL performed the bioinformatic analysis. JQ and QL collected, evaluated the clinical, and genetic evidence. LS and JL did the functional studies on zebrafish model. QT revised the manuscript. All authors read and approved the final manuscript.

## Conflict of Interest

JL was employed by the company Cipher Gene LLC. WG and PW were employed by the company Chigene (Beijing) Translational Medical Research Center Co., Ltd. The remaining authors declare that the research was conducted in the absence of any commercial or financial relationships that could be construed as a potential conflict of interest.

## Publisher’s Note

All claims expressed in this article are solely those of the authors and do not necessarily represent those of their affiliated organizations, or those of the publisher, the editors and the reviewers. Any product that may be evaluated in this article, or claim that may be made by its manufacturer, is not guaranteed or endorsed by the publisher.

## Footnotes

1
http://www.brainspan.org/

2
www.r-project.org/
